# Animal-human interfaces and emerging zoonoses: *Salmonella* in pet turtles in Concepción, Chile

**DOI:** 10.3389/fvets.2026.1783800

**Published:** 2026-06-04

**Authors:** Felipe Faundez, Hernan Cañon-Jones, Karen Fehrmann-Cartes

**Affiliations:** Escuela de Medicina Veterinaria, Facultad de Medicina Veterinaria y Agronomía, Universidad de Las Américas, Santiago, Chile

**Keywords:** One Health, public health, reptile, salmonellosis, zoonosis

## Abstract

*Salmonella*, a globally distributed genus of *Enterobacteriaceae*, poses a public health risk due to its role in gastrointestinal and systemic infections. Red-eared slider turtles (*Trachemys scripta elegans*) are asymptomatic carriers of various *Salmonella* serovars. The objective of this study was to study the prevalence of *Salmonella* in different parts of pet turtles and their environment. In total, 179 samples (cloacal, shell swabs, and water) were collected from 72 pet turtles in Concepción, Chile. Using selective media and biochemical tests, 50 presumptive *Salmonella* isolates were found in 42 turtles, indicating a 58.3% prevalence. Shell swabs had the highest positive prevalence (30.6%), followed by water (28.6%) and cloacal samples (25.0%). The high contamination on shell surfaces suggests a direct fomite route for zoonotic transmission. These findings confirm pet turtles as significant *Salmonella* reservoirs and highlight the need for One Health strategies integrating veterinary care, public health education, and environmental hygiene to reduce salmonellosis risk.

## 1 Introduction

The frequency and intensity of interactions at the human-animal-environment interface have expanded dramatically in the 21st century, driven by globalization, the exotic pet trade, urbanization, and changes in land use (1, 2). These interfaces are the critical crossroads for the emergence and spillover of zoonotic pathogens, which constitute approximately 60% of known infectious diseases in humans and up to 75% of new or emerging infectious diseases (3, 4). The COVID-19 pandemic serves as a recent reminder of the global devastation that can originate from a zoonotic event, emphasizing the urgent need for proactive surveillance and a holistic understanding of disease dynamics (2, 5). The One Health approach has emerged as an essential paradigm. It is defined as the collaborative, multisectoral, and transdisciplinary effort to achieve optimal health outcomes by recognizing the interconnection between people, animals, plants, and their shared environment, providing the foundational framework for addressing these complex challenges (6, 7). This is particularly relevant for zoonotic diseases like salmonellosis, where transmission cycles intricately involve animal reservoirs, environmental contamination, and human behavior. Salmonellosis, caused by Gram-negative bacteria of the genus *Salmonella*, remains a major global foodborne and direct-contact zoonosis, with an estimated 93.8 million human cases and 155,000 deaths annually worldwide (8). While traditionally associated with poultry, swine, and cattle, reptiles are now well-established asymptomatic reservoirs of a vast diversity of *Salmonella* serovars, many of which are pathogenic to humans (9, 10). The popularity of reptiles, particularly red-eared sliders (*Trachemys scripta elegans*), as pets brings this wildlife reservoir into intimate contact with humans, especially children, creating a high-risk interface for zoonotic transmission (11). These animals can shed the bacterium intermittently in their feces, contaminating their skin, shell and the aquatic environment in which they live. Humans typically acquire the infection through the fecal-oral route, often via hand-to-mouth contact after handling the animal or contaminated objects (tanks, decorations or water) without proper hygiene (12, 13). The public health significance of this issue was recognized decades ago. In the 1970s, the United States implemented a ban on the sale of turtles with a carapace length of less than 4 inches due to a significant public health burden linked to pet turtle-associated salmonellosis, particularly in children. This measure has proven effective, yet it is not universally adopted, and illegal sales persist in the US, reflecting the potential higher risk of infections due to uncontrolled or untraceable sales (14, 15), while in Chile there is no information in this issue. It is estimated that reptiles are responsible for 6% to 11% of all sporadic *Salmonella* infections in humans in the United States (approximately 74,000 cases annually) and for an estimated 93,000 cases each year in Colombia (16, 17). In Chile, a retrospective study of reptiles imported between 1997 and 2008 found a startling 70.3% prevalence of *Salmonella* upon quarantine (18), but data on the prevalence in established pet populations within households is scarce. Furthermore, the overuse and misuse of antibiotics in human and veterinary medicine have increased the emergence of multidrug-resistant (MDR) *Salmonella* strains. These MDR strains have been increasingly reported in reptiles, including turtles, complicating treatment options and posing a grave public health threat (19, 20). Factors such as overcrowding, inadequate temperature and humidity, poor sanitation, transport, and excessive handling can stress reptiles, potentially trigger increased excretion of the bacteria and amplify the risk of transmission (12). The red-eared slider turtle is among the most common pet reptiles globally, and pathogenic *Salmonella* has been detected in captive populations in Spain (12, 21), China (20), and Japan (22). In Chile, studies have been conducted in sliders in quarantines facilities or studies in pet turtles but using only cloacal samples (18, 23). However, no contemporary studies have explored *Salmonella* presence in pet red-eared turtles in Chile using a multi-sample approach. Thus, given the importance of this infectious agent in public health and the high probability of contagion through turtles due to improper management. The objective of this study was to detect *Salmonella* spp. in *Trachemys scripta elegans* from pet stores and owned as pets in Chile by analyzing cloacal, shell and environmental water samples to descriptively document the occurrence of *Salmonella* and assess relative prevalence from different sources. By doing so, this research aims to highlight a potential public health risk often neglected in Chile, and globally, and to guide future research and surveillance efforts.

## 2 Materials and methods

### 2.1 Study area

The study was conducted in Concepción city, the third largest city in Chile, located in the Bío Bío region of central Chile. This area is a major urban city with a high population density, and where many households may kep exotic pets. The study protocol was reviewed and approved by the Bioethics Committee of the Faculty of Veterinary Sciences of the University of Concepción, ensuring all procedures adhered to ethical standards for animal handling and sample collection in Chile.

### 2.2 Sample size and collection

The sample size was calculated assuming 3% of pet ownership for red eared turtles in Concepción, with 95% level of confidence, 10% margin of error, and 75% response distribution. Red-eared turtles (*Trachemys scripta elegans*), of any sex and age, housed individually, in pairs, or in groups within aquariums, were included in the study. A total of 72 red-eared slider turtles (*Trachemys scripta elegans*) were selected for this study with a total of 179 samples collected and analyzed (72 cloacal swabs, 72 carapace swabs, and 35 water samples from the respective shared aquariums). The sample population included turtles from two primary sources: specialized pet stores (representing animals for sale) and private owners (representing household pets). The cohort included both males and females of varying ages and sizes, living under different husbandry conditions: individually, in pairs, or in groups within their aquariums or tanks. For each animal (or shared aquarium), three distinct types of samples were collected to maximize detection sensitivity: (a) Cloacal swab: to detect active intestinal carriage and shedding, (b) Shell swab: to detect environmental contamination of the turtle's surface, acting as a fomite, and (c) Water sample: to detect contamination of the shared aquatic environment. Rigorous biosecurity measures were enforced throughout the sampling process to prevent zoonotic transmission and cross-contamination. Personnel wore disposable gloves for each turtle handling event. All equipment and surfaces that came into contact with the turtles or their aquaria were disinfected with 70% ethanol between samplings. Turtles were gently restrained by holding the plastron and the edges of the carapace to minimize stress. For the cloacal swabs, the tail was carefully lifted, and a sterile BBL CultureSwab was inserted into the cloaca, using gentle rotational movements to collect fecal material or mucosal cells. For very small juveniles where cloacal insertion was not feasible, the sample was collected from the pericloacal mucosa. Shell swab samples were obtained using a new sterile BBL CultureSwab and the entire dorsal surface of the carapace was thoroughly rubbed, focusing on areas likely to hold contamination. Water samples were obtained by collecting approximately 10 to 15 mL of water aseptically collected into a sterile, leak-proof container. Any anecdotal observation between husbandry conditions and positivity was recorded.

### 2.3 Laboratory analyses

All collected samples were clearly labeled with a unique identifier and immediately place in a cooler with frozen gel-packs to maintain a temperature between 4 °C and 8 °C. Transport to the laboratory was expedited (1 to 2 h) and all analyses were initiated within 24 h of collection to ensure sample integrity. All samples were processed at the Microbiology Laboratory of the Department of Pathology and Preventive Medicine, Faculty of Veterinary Sciences, University of Concepción, Chillán Campus. The analytical protocol followed established methods for the isolation and identification of *Salmonella* (24, 25) using a multiphase method (pre-enrichment, selective enrichment and selective plating, and biochemical analysis).

### 2.4 Statistical analyses

Descriptive statistics were used to calculate overall individual prevalence, prevalence by sample type (cloacal, shell, and water) and prevalence by source (pet store vs. private owner). Chi-squared test were used to describe any differences between positivity of samples. Data were tabulated and analyzed using Jamovi. Results are presented as counts and percentages.

## 3 Results

### 3.1 Individual turtle positivity rate

The individual-level prevalence of presumptive *Salmonella* carriage was 58.3% (42/72).

### 3.2 Positivity rate by sample type

50 samples from all types of samples (*n* = 179) were positive for *Salmonella* spp., yielding an overall sample positivity rate of 27.9%, as can be seen in Table 1. The shell swabs yielded the highest number of positive samples (*n* = 22, 30.6%), followed closely by the water samples from the aquariums (*n* = 10, 28.6%). Cloacal swabs had the lowest positivity rate among the three sample types (*n* = 18, 25.0%).

**Table 1:** Accumulated frequency of *Salmonella spp*. detection by sample type.

Sample type	Positive (*n*, %)	Negative (*n*, %)	Total
Cloacal swabs	18 (25.0%)	54 (75.0%)	72
Shell swabs	22 (30.6%)	50 (69.4%)	72
Aquarium water	10 (28.6%)	25 (71.4%)	35^*^

^*^Turtles sharing aquarium.

### 3.3 Observations on husbandry and positivity

However, no differences existed between sample types (*X*^2^= 0.40, *df* = 2, *p* = 0.819) or source (*X*^2^= 0.208, *gl* = 1, *p* = 0.901). Anecdotal observations during sampling suggested a potential correlation between husbandry conditions and positivity. Turtles kept in tanks with visibly dirty water, overcrowded conditions, or inadequate filtration systems appeared be related to positive samples, particularly from the shell and water. However, this variable was not quantitatively measured in this study.

## 4 Discussion

This study reveals a high positivity rate (58.3%) of *Salmonella* carriage in individual pet red-eared slider turtles in Concepción, Chile. The detection rate is high and reveals the role of these popular pets as significant asymptomatic reservoirs of a major zoonotic pathogen, creating a direct and often overlooked public health risk at the human-animal interface. This positivity rate of 58.3% found in this study is substantially higher than the 13% reported in a previous investigation in Valdivia, Chile (23). This discrepancy can be largely attributed to the comprehensive sampling methodology employed in our study. While the Valdivia study relied solely on cloacal swabs, our inclusion of shell and environmental water samples proved critical for detection. The fact that shell swabs yielded the highest individual positivity rate (30.6%) is one of the most significant public health findings. It clearly demonstrates that the risk of transmission extends far beyond contact with feces. The turtle's shell acts as a constant fomite, contaminated either directly by feces or by the contaminated aquatic environment. Handling the turtle, cleaning the tank, or even touching the water can thus serve as exposure routes, especially for children who may touch the turtle and then their mouths or faces (11, 13).

The lower positivity rate in cloacal samples (25%) is consistent with the well-documented phenomenon of intermittent shedding in reptiles (12, 26). *Salmonella* can colonize the intestinal tract without being constantly excreted in feces. This intermittency means that a single negative cloacal test cannot rule out *Salmonella* carriage, revealing a major limitation of relying on fecal testing alone and unequivocally validating the necessity of our multi-sample approach for accurate risk assessment.

The positivity in water samples (28.6%) further confirms the extent of environmental contamination. The aquarium water serves as a breeding ground and a persistent reservoir for the bacteria, continually re-contaminating the turtles and anything that comes into contact with the water (27). This creates a cycle of infection within the tank that can persist even if an individual turtle is not actively shedding at a given moment.

The high positivity rate we report is consistent with the 70.3% found in a retrospective study of imported reptiles at a Chilean quarantine station between 1997 and 2008 (18). The difference between our finding (58.3%) and the quarantine data can be logically explained by the “pooling” effect and the extreme stress associated with quarantine. In quarantine settings, hundreds of animals are often housed in proximity, and the status of an entire group may be considered positive based on a single positive animal. Furthermore, the immense stress of capture, international transport, and high-density housing in quarantine are known immunosuppressants that can exacerbate bacterial shedding (26, 28). In contrast, pets in home aquariums, while often kept in suboptimal conditions, likely experience lower overall stress levels, potentially leading to the lower (though still very high) prevalence we observed.

Internationally, our results align with studies reporting high carriage rates in pet turtles. For example, a 27% positivity in fecal samples from semi-aquatic turtles in Colombia (17). A study in Spain detected *Salmonella* in 15% of captive turtles, with intestinal content being the best sample (21). Studies in free-ranging turtle populations in Texas found a 36.5% prevalence using PCR, suggest that environmental factors and cross-species contamination can drive prevalence even higher (29). Given the high prevalence of *Salmonella* in turtles in this study, reptile-associated salmonellosis should be considered a potential public health concern and further investigation should be conducted.

The public health implications of our findings are profound. The Center for Disease Control and Prevention (CDC) and other health agencies consistently warn that reptiles are inappropriate pets for households with children under 5, the elderly, pregnant women or immunocompromised individuals due to the risk of salmonellosis (13, 30). Yet, these animals remain widely available and popular. The lack of knowledge among pet owners is a critical risk factor. No studies have been conducted in pet turtle owner in Chile, but based in studies elsewhere, many owners are completely unaware that their apparently healthy turtle can carry dangerous pathogens (31).

This study had some limitations and results should be taken with care. The aim of the study was to detect the presence of *Salmonella* in turtles in Concepcion, Chile, and the authors acknowledge that these are preliminary results. The use of biochemical tests alone, while standard, provides only identification to the genus level and cannot identify specific serovars or their pathogenicity. Future work should incorporate serotyping and molecular techniques (e.g., PCR for the invA or hilA genes) to identify the specific *Salmonella* serovars present and assess their potential to cause human disease (32). Furthermore, investigating the antimicrobial resistance profiles of these isolates is critical given the global rise of MDR *Salmonella* (21, 22).

Future studies should also quantitatively assess husbandry practices (e.g., water change frequency, tank size, cleaning protocols, and number of turtles per aquarium) to statistically correlate them with *Salmonella* prevalence and bacterial load, as noted in the results. Future research could also explore the efficacy of various disinfectants against *Salmonella* in aquarium environments.

This situation demands a robust One Health response. Mitigation requires coordinated action across multiple sectors such as (a) Veterinarians: Veterinarians play a crucial role in educating clients about the risks of reptile ownership during pre-purchase consultations and welfare examinations. They should advocate for proper husbandry (adequate tank size, good filtration and cleaning practices) to reduce bacterial load and stress, (b) Public Health: Public health agencies should develop and disseminate clear, accessible educational materials in pet stores, clinics, and schools. Messaging must emphasize that “all reptiles can carry *Salmonella*” and stress the non-negotiable necessity of rigorous handwashing with soap and water immediately after handling turtles or anything in their habitat, (c) Policy and regulation: There is a need to review and potentially strengthen regulations regarding the sale of pet reptiles. This could include mandatory point-of-sale information sheets and warnings, similar to those used in some other countries.

## 5 Conclusion

This study detected presumptive *Salmonella* spp. in 58.3% of pet red-eared slider turtles (*Trachemys scripta elegans*) in Concepción, Chile, with shell swabs showing the highest contamination rate (30.6%). These findings should be considered as starting point of research, as identification was based on biochemical methods without serotyping or molecular confirmation. Nevertheless, the results provide valuable baseline data on *Salmonella* carriage in pet turtles in Chile, a topic for which contemporary information is limited. The multi-sample approach (including cloacal, shell, and environmental water) proved useful for detecting contamination across different reservoirs, with shell surface positivity highlighting a potential direct route for zoonotic transmission through handling. Also, our results showed that collecting cloacal swabs may not be the most adequate for surveillance. These preliminary findings support the need for larger, confirmatory studies incorporating serotyping and antimicrobial resistance profiling. They also reinforce the importance of public health messaging regarding hygiene practices when handling pet reptiles, particularly in households with children.

## Data Availability

The original contributions presented in the study are included in the article/Supplementary material, further inquiries can be directed to the corresponding author.
